# Development and Validation of an Ultrasensitive Procalcitonin Sandwich Immunoassay

**DOI:** 10.3390/ht6040018

**Published:** 2017-11-16

**Authors:** Viviana A. Carcamo Yañez, Jens C. Göpfert, Markus Otto, Hayrettin Tumani, Andreas Peter, Thomas O. Joos

**Affiliations:** 1 NMI Natural and Medical Sciences Institute at the University of Tuebingen, 72770 Reutlingen, Germany; viviana.carcamo@nmi.de (V.A.C.Y.); jens.goepfert@nmi.de (J.C.G.); 2 Department of Neurology, University of Ulm, 89081 Ulm, Germany; markus.otto@uni-ulm.de (M.O.); hayrettin.tumani@uni-ulm.de (H.T.); 3 German Centre for Diabetes Research (DZD), 72076 Tübingen, Germany; Andreas.Peter@med.uni-tuebingen.de; 4 Institute for Diabetes Research and Metabolic Diseases of the Helmholtz Centre Munich at the University of Tübingen, 72076 Tübingen, Germany; 5 Department of Internal Medicine, Division of Endocrinology, Diabetology, Angiology, Nephrology and Clinical Chemistry, Tübingen University Hospital, 72076 Tübingen, Germany

**Keywords:** single molecule array, validation, procalcitonin, base line level

## Abstract

Procalcitonin (PCT) is well established as a highly specific biomarker for the detection of bacterial infections and sepsis. However, the currently available diagnostic tests are not able to detect very low or very early increases of PCT or even baseline levels in healthy individuals or patients with non-bacterial infections. In order to be able to detect these very low concentrations of PCT, a sandwich immunoassay was developed using high sensitivity Single Molecule Array technology (Simoa). The assay was thoroughly validated and applied to analyze human cerebrospinal fluid (CSF) and serum samples from patients with bacterial or viral meningitis as well as CSF, serum, and K2 EDTA plasma from healthy control subjects. A 50-fold increase in sensitivity compared to the current gold standard assays was achieved, which was sensitive enough for the detection of baseline PCT levels. Both serum and CSF showed significantly elevated PCT levels in patients with bacterial meningitis compared to patients with viral meningitis and the healthy control group. Procalcitonin concentration levels for patients with viral meningitis and the control group could be measured, but were not significantly different. The determination of PCT in the low pg·mL^−1^ range could help to improve the monitoring of bacterial infectious diseases, as PCT level changes could be detected earlier.

## 1. Introduction

Procalcitonin (PCT) is a well-established biomarker for the clinical diagnosis of bacterial infections [1,2,3,4]. Procalcitonin is a precursor of the hormone calcitonin and consists of 116-amino acids, which are normally processed by a specific protease to calcitonin, katacalcin, and an N-terminal residue [5]. In healthy individuals, PCT levels are very low and not quantifiable with conventional ligand binding assays. During bacterial infections or septic conditions, the concentration of PCT can increase up to 1000 ng·mL^−1^; this makes PCT a very valuable biomarker for detecting bacterial infections, such as bacterial sepsis, blood stream infections, and infection of the respiratory tract or several other human organs (recently reviewed by Sager et al. [6]). Procalcitonin analysis was also reported to be a valuable marker for the selection of antibiotics for the treatment of bacterial infections. It can also help to reduce antibiotic prescription rates and to evaluate the duration of antibiotic use [6].

The available diagnostic assays perform well within the currently targeted clinically relevant PCT concentration range. Several of the commercially available PCT assays on various clinical platforms are based on identical reagents from a single provider, and detection limits vary with the applied immunoassay technology. Some companies have meanwhile also developed point-of-care devices for the detection of elevated PCT levels (nicely summarized in Schuetz et al. [7]). However, in all of these assays, PCT baseline levels are below the quantification limit of these assays. With the recent developments in ultrasensitive immunoassay technologies, it becomes possible to measure protein concentrations below the quantification levels of the well-established assay technologies used in routine analysis in the research and diagnostics of analytes from human body fluids.

One of these new highly sensitive immunoassay technologies is the single molecule array (Simoa) platform, which allows for the measurement of proteins down to the low femtomolar range [8,9,10,11,12]. The Simoa technology is able to detect single enzyme-labeled immunocomplexes using femtoliter-sized wells for readout. Immunocomplexes are formed on paramagnetic microparticles with immobilized capture antibodies and a biotinylated detection antibody. After capturing the target protein and binding the second detection antibody, the immunocomplexes are labeled with an enzyme using the enzyme conjugate streptavidin-β-galactosidase (SβG). The detection of these enzyme-labeled immunocomplexes is performed using the galactosidase substrate resorufin β-d galactopyranoside (RGP). The substrate is added to the microparticles, and the microparticles–substrate mix is then transferred onto an array of femtoliter-sized wells, which are able to hold only one microparticle. After loading the microparticles, the array is sealed with oil. Because of the femtoliter-sized wells, the enzymatic activity (cleavage of RGP) of one single immunocomplex releases enough fluorescence product, resorufin, to be detected by the charge coupled device (CCD) camera of the analyzer. The combination of a microparticle-based immunoassay with an Enzyme-linked Immunosorbent Assay (ELISA)-like enzymatic signal amplification results in highly sensitive immunoassays, which outperform traditional immunoassay techniques in terms of sensitivity by 50–500 fold. More details and schematic overviews of the Simoa technology are provided in the available literature [8,9,10,11,12]. We have developed an ultrasensitive sandwich immunoassay for procalcitonin using the Simoa technology. This assay format increased PCT detection by a factor >50 compared to the currently available technologies. This assay was used for the analysis of human cerebrospinal fluid (CSF) and serum samples from patients with bacterial and viral meningitis together with appropriate control samples. Our aim was to determine the PCT baseline level in healthy individuals and to explore if a more sensitive PCT immunoassay could improve the discrimination of patients suffering from viral or bacterial meningitis.

## 2. Materials and Methods

### 2.1. Preparation of Capture and Detection Antibody

Capture antibody (rat-anti human CALCA, clone 4A6) and detection antibody (rat-anti human CALCA, clone 42F3) for the development of the Simoa Procalcitonin sandwich immunoassay were provided by Helmholtz Zentrum München (Munich, Germany).

The capture antibody was immobilized on paramagnetic microparticles using a two-step 1-ethyl-3-(3-dimethylaminopropyl) carbodiimide hydrochloride (EDC) (Thermo Fisher Scientific, Waltham, MA, USA) and sulfo-*N*-hydroxysulfosuccinimide (sulfo-NHS) (Thermo Fisher Scientific) coupling reaction. To remove the capture antibody storage buffer, a buffer exchange was carried out with Amicon 50 kDa spin filters (Merck, Darmstadt, Germany) following the manufacturer’s recommendations. During this procedure, the antibody storage buffer was replaced by Bead Conjugation Buffer (Quanterix, Lexington, MA, USA). The concentration of the recovered antibody was determined at 280 nm using a spectrophotometer (NanoDrop 2000, Thermo Fisher Scientific). The antibody concentration was adjusted to 0.5 mg·mL^−1^ with Bead Conjugation Buffer and stored at 4 °C. A total of 2.8 × 10^8^ paramagnetic carboxylated microparticles (Quanterix) were transferred into a 0.5 mL low protein binding reaction tube and washed three times with 200 µL Bead Wash Buffer (Quanterix) and two times with Bead Conjugation Buffer using a magnetic separator. After the washing steps, the supernatant was removed and the beads were re-suspended in 160 µL of Bead Conjugation Buffer. To activate the microparticles, 20 µL of the EDC (50 mg·mL^−1^) and 20 µL of the sulfo-NHS solution (50 mg·mL^−1^) were added to the microparticles solution to reach a final EDC and sulfo-NHS concentration of 5 mg·mL^−1^. The microparticles were mixed and placed on a rotator (HulaMixer, Thermo Fisher Scientific) for 30 min at room temperature (RT). After activation, the microparticles were washed with 200 µL of Bead Conjugation Buffer using a magnetic separator. The supernatant was removed and 200 µL of the prepared capture antibody solution was added to the microparticles. The microparticles–antibody solution was mixed and placed on a rotator for 120 min at room temperature. After antibody conjugation, the microparticles were washed twice with 200 µL of Bead Wash Buffer (Quanterix). The microparticles were blocked on a rotator for 30 min at room temperature with Bead Blocking Buffer (Quanterix). After incubation, the microparticles were washed with 200 µL of Bead Wash Buffer followed by a wash step with 200 µL of Bead Diluent (Quanterix). In a final step, the microparticles were re-suspended in 200 µL of Bead Diluent.

The rat-α-hu PCT clone 2F3 detection antibody was biotinylated using NHS-PEG4-Biotin (Thermo Fisher Scientific). The antibody was diluted in Biotinylation Reaction Buffer (Quanterix) to a final concentration of 1.0 mg·mL^−1^ in 2 mg of NHS-PEG4-Biotin that was reconstituted in 1 mL double distilled water (ddH_2_O) just prior to use. To start the biotinylation reaction, 4.5 µL of the biotin solution were added to 100 µL of the antibody solution, vortexed, briefly centrifuged, and incubated for 30 min at room temperature. The final molar biotin challenge ratio was 23. To remove the unbound biotin, the antibody–biotin solution was transferred to an Amicon 50 kDa spin filter and a buffer exchange was carried out with Biotinylation Reaction Buffer following the manufacturer’s recommendations. The concentration of the recovered antibody was determined at 280 nm using a spectrophotometer and adjusted to 0.2 mg·mL^−1^ with Biotinylation Reaction Buffer. Before storage at −20 °C, the antibody solution was diluted 1:2 in glycerol (Sigma Aldrich, St. Louis, MO, USA).

### 2.2. Simoa Assay Setup and Preparation of Reagents

All Simoa measurements were performed on a fully automated Simoa HD-1 Analyzer (Quanterix). The microparticles, coated with PCT capture antibody, were diluted in Bead Diluent (Quanterix) 1:100. The number of active beads was reduced and replaced by non-reactive microparticles (Helper Beads, Quanterix). Approximately 350,000 non-functional and 350,000 functional microparticles per sample were used. The PCT detection antibody was diluted in Detector Diluent (Quanterix) to a working concentration of 1.0 µg·mL^−1^. Streptavidin-β-galactosidase concentrate was diluted in SβG Diluent (Quanterix) to a working concentration of 150 pM. Resorufin β-d-galactopyranoside substrate was used as provided by Quanterix. All prepared samples, calibrators, and serum/CSF samples were transferred into a 96-well plate (Quanterix) for the measurement. The assay configuration protocol was a three-step assay. In the first step, 25 µL of the microparticles solution and a 75 µL sample were incubated for 30 min (40 cadences) in a reaction cuvette (Quanterix), followed by several wash steps. In the second step, 100 µL of detector antibody was added to the microparticles and incubated for 5 min and 15 s (7 cadences), followed by several wash steps. In the third step, 100 µL of SβG was added and incubated for 5 min and 15 s (7 cadences), followed by several wash steps. Twenty microliters of RGP substrate solution were added to the microparticles, mixed, and loaded onto the Simoa disc array. The array was then sealed with oil and microparticles were imaged. Automated analysis was done by the HD-1 Analyzer software version 1.5 (Quanterix).

### 2.3. Simoa Assay Validation Procedure

During assay validation, the following basic assay parameters were addressed: calibration curve model, limits of quantification (upper limit of quantification (ULoQ) and lower limit of quantification (LLoQ)), sensitivity, precision (intra- and inter-assay), spike-in recovery, dilutional linearity, parallelism, and analyte stability (freeze-thaw, benchtop). Validation was done using a fit-for-purpose approach [13,14] and also considering commonly used guidelines from health authorities (e.g., European Medicines Agency (EMA), Food and Drug Administration (FDA)) [15,16] as far as applicable to ligand–binding assays.

The validation of the calibration curve model was done by running eight independent experiments including the calibration curve in duplicates. The coefficient of variation (CV) was determined over all assay runs using the recalculated concentration values. To generate the calibration curve, recombinant human PCT (Fitzgerald Industries International, Acton, MA, USA) was serially diluted, and the final concentrations of the calibrators in the assay were 1.23 to 900 pg·mL^−1^. The calibrator/sample diluent was PBS supplemented with 4% (*w/v*) bovine serum albumin (Merck), 0.25% Tween-20 (Sigma Aldrich), and 0.3% (*w/v*) K2 EDTA (Sigma Aldrich). Acceptance criteria were a recovery within 80–120% and a CV below 20% of all back-calculated calibrator samples. The LLoQ was determined by two-fold dilutions of standard four (33.3 pg·mL^−1^) of the calibrator curve for six dilutions in calibrator/sample diluent. The concentration of the last LLoQ determination sample was 0.52 pg·mL^−1^. The samples were measured in triplicates over three independent assay runs on three different days. The LLoQ was defined as the point at which the CV was below 20% and recovery was still within 80–120% of the nominal concentration. The upper limit of quantification (ULoQ) was established after six assay runs using the results generated from the calibrator curve. The highest calibrator concentration where recovery was within 70–120% and the CV below 20% was considered as the ULoQ. The assay’s sensitivity was determined by measuring >20 replicates of the zero calibrator (calibrator/sample diluent) in one assay run. The mean raw data of the zero calibrator and the concentration of the two next calibrator points were calculated, and a line fit was used to determine the concentration of the average zero calibrator plus 3× SD. Intra-assay precision was determined in the high, mid, and low assay range by measuring spiked or native human EDTA plasma in 12 replicates each within one run. The spike-in concentration for the high intra-assay sample was 300 pg·mL^−1^, and for the mid/low intra-assay sample 10 pg·mL^−1^. As, in the low intra-assay sample, native human EDTA plasma was used, the intra-assay precision in the mid-range of the assay was not supposed to exceed a CV of 15% and 20% at the range of the ULoQ and LLoQ, respectively. Inter-assay precision was determined in the high, mid, and low assay range by measuring three spiked and two native human EDTA plasma samples in triplicates over eight assay runs on five different days. The spike-in concentrations for the high inter-assay samples were 300 pg·mL^−1^ and 90 pg·mL^−1^, and for the mid/low inter-assay sample 10 pg·mL^−1^. For determination in the low assay range, two native human EDTA plasma samples were used. Inter-assay precision was not supposed to exceed a CV of 20%. All intra and inter-assay samples were diluted by the factor 7.5 with calibrator/sample diluent prior to measurement. Due to the dilution of the samples with microparticles solution during sample processing in the HD-1 Analyzer, the final sample dilution factor was 10. Recovery was determined in the high, mid, and low assay range by spiking human EDTA plasma, with a very low endogenous PCT level, with different amounts of recombinant calibrator protein. Measurements were done in triplicates within one run. Acceptable performance was a recovery within 80–120% maintained over the working range of the assay. To determine the dilutional linearity, human EDTA plasma was spiked with 5000 pg·mL^−1^ calibrator protein and serially diluted throughout the assay range with calibrator/sample diluent. The spiked sample was first diluted by 1:8 to bring the analyte concentration into the assay range, then further diluted serially by 2.5-fold dilutions. Measurements were done in triplicates within one run. Acceptance criteria were a recovery within 80–120% of the nominal concentration maintained over the working range. Parallelism was determined by serial two-fold dilutions of a human EDTA plasma sample with a high endogenous PCT level, for six dilutions. Measurements were done in triplicates within one run. Acceptance criteria were a recovery within 80% and 120%, and the average of all recalculated concentrations was considered the 100% reference value. Analyte stability was addressed by performing freeze-thaw and short-term stability tests. A human EDTA plasma sample was spiked with 10 pg·mL^−1^ recombinant human PCT, aliquoted into 10 identical aliquots, and immediately frozen at −80 °C. To demonstrate the analyte stability during repeated freeze–thaw cycles, three of the prepared samples were thawed at room temperature and frozen at −80 °C repetitively to obtain samples with one to three additional freeze–thaw cycles. To determine the short-term temperature stability, six of the prepared samples were stored at 2–4 °C or at room temperature for up to 24 h. The samples were thawed and incubated at the according temperature and time prior to the start of the assay processing. All stability samples were measured in triplicates and compared to the concentration of a fresh control sample. Acceptance criteria were a recovery within 80% and 120%, and the back-calculated concentration of the control sample was considered the 100% reference value.

### 2.4. Laboratory Methods

Currently, the B·R·A·H·M·S PCT sensitive KRYPTOR assay (Thermo Scientific, Hennigsdorf, Germany) is considered the “gold standard” for PCT measurements in a clinical setting, and has been used to develop clinical cut-offs and algorithms. The KRYPTOR random access analyzer for homogeneous immunoassays in human serum or plasma uses the time-resolved amplified cryptate emission (TRACE) technology. It has a functional assay sensitivity (LLoQ) for PCT of 0.06 μg·L^−1^. All KRYPTOR measurements were performed at the Central Laboratory of University of Tübingen Medical Center. The Laboratory holds an accreditation according to ISO 15189, and internal as well as external quality controls for PCT were within the allowed ranges at all times.

### 2.5. Human Blood and CSF Samples

Eighteen human serum and twenty four EDTA plasma (K2) samples from male donors and twenty three serum samples and twenty five EDTA plasma (K2) samples from female donors, of different ages and races, were purchased from Seralab Laboratories International LTD (West Sussex, UK). Information about the donors is presented in Table S2. All serum and EDTA plasma samples were recovered from whole blood donations. Samples were thawed at 4 °C after being received from the vendor, aliquoted, and stored at −80 °C. All of these samples were diluted by the factor 4.5 with calibrator/sample diluent prior to measurement. Due to the dilution of the samples with microparticles solution during sample processing in the HD-1 Analyzer, the final sample dilution factor was 6. Measurements were done in duplicates.

Serum and cerebrospinal fluid samples of fifteen patients (age: 47 years, standard deviation (SD): 17 years) with diagnosed bacterial meningitis (BM), fifteen patients (age: 50 years, SD: 19 years) with diagnosed viral meningitis (VB), and fifteen patients without any clinical sign of an inflammatory process in the brain (controls) (age: 51 years, SD: 19 years) were contributed by the University of Ulm, Department of Neurology (Ethical approval number 20/10) (Table S1). Samples were diluted by the factor 7.5 with calibrator/sample diluent prior to measurement, resulting in a final dilution factor of 10. Measurements were performed in duplicates.

## 3. Results and Discussion

### 3.1. Assay Development and Validation

For the measurement of human PCT in the lower pg·mL^−1^ range, a bead-based immunoassay was developed using the Simoa technology (Quanterix). The immunoassay development process included the evaluation of a suitable antibody pair and the optimization of assay conditions, such as applied reagent concentrations, assay buffer composition, and incubation times.

Two monoclonal rat antibodies directed against human Procalcitonin (CALCA, clone 4A6 and 2F3, Helmholtz Zentrum München) were tested as capture and detection antibodies. These antibodies were selected as a matching pair with high affinity for PCT with no cross-reactivities to calcitonin and katacalcin in sandwich immunoassays [17]. When tested as capture reagents, the antibodies were immobilized on paramagnetic microparticles, and when applied as detector reagents, the antibodies were biotinylated following standard procedures. Recombinant human PCT (Fitzgerald Industries International) was used as calibrator protein. The best assay performance was achieved when CALCA clone 4A6 was used as the capture antibody and clone 2F3 was used as the detection antibody. CALCA clone 4A6 is directed against the pro-peptide of PCT, CALCA 2F3, against the calcitonin part [17]. Therefore, the antibody clone 4A6 detects PCT with higher specificity compared to clone 2F3. Various assay conditions were tested (results not shown). The best assay performance, evaluated based on the calibrator curve, was obtained in a three-step assay with a detector antibody concentration of 1 µg·mL^−1^ (100 µL volume) and a SβG concentration of 150 pM (100 µL volume). Twenty-five microliters of microparticles (350,000 active and 350,000 inactive microparticles) and 75 µL of sample were used. The calibrator/sample diluent was PBS supplemented with 4% (*w*/*v*) bovine serum albumin, 0.25% Tween-20, and 0.3% EDTA.

**Figure 1:**
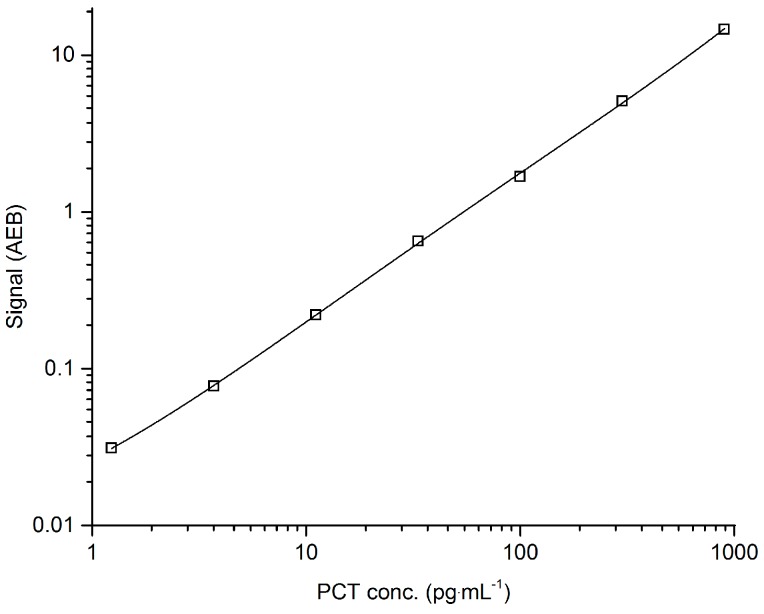
Typical Simoa Procalcitonin (PCT) assay calibration curve. Serially diluted recombinant human PCT. The calibrator range was 1.23 to 900 pg·mL^−1^ with a recovery of all back-calculated concentrations between 99% and 101%. The fitting model for the calibration curve was a weighted four-parameter logistics (1/Y^2^). AEB: Average enzyme per bead (measured signal).

During assay validation, the following basic assay parameters were addressed: calibration curve model, limits of quantification (ULoQ and LLoQ), sensitivity, precision (intra- and inter-assay), spike-in recovery, dilutional linearity, parallelism, and analyte stability (benchtop, freeze-thaw). The fitting model for the calibration curve was a weighted four-parameter logistics (1/Y^2^). The recovery of all back-calculated concentrations of the individual calibrator points was between 99% and 101%. The CV for all back-concentrations of the individual calibrators was below 10%. A typical Simoa PCT immunoassay calibration curve is given in Figure 1.

The assessment of sensitivity and assay dynamic range revealed a limit of detection (LoD) of 0.44 pg·mL^−1^, and LLoQ and ULoQ of 1.23 and 900 pg·mL^−1^, respectively. Intra-assay precision in the low, mid, and high assay range (three levels, *n* = 12 each) resulted in CVs between 12.9% and 15.3%. Inter-assay precision in the low, mid, and high assay range (five levels, over eight independent runs) resulted in CVs between 10.7% and 19.4%. Spike-in recovery in the low, mid, and high assay range (three levels) was between 90% and 99%. Dilutional linearity of a spiked sample showed a recovery between 82% and 117% over the working range when diluted 8–4883-fold, respectively. Parallelism of a native sample showed a recovery of between 91% and 115% when diluted 2–64-fold, respectively. For the dilutional linearity and parallelism experiments, the average of the recalculated concentrations was considered to be the nominal value for the recovery calculations. Analyte stability was given for at least three freeze-thaw cycles, and recovery was between 97% and 104%. Short-term analyte stability was given for samples stored at room temperature for up to 24 h, and recovery was between 83% and 105%. All of the validation acceptance criteria and the obtained results are shown in Table 1. 

The developed Simoa PCT assay did fulfill acceptance criteria for all addressed validation parameters considered in the commonly used guidelines from health authorities (e.g., EMA, FDA) as far as applicable to ligand-binding assays. The method validation demonstrated that the required precision and reliability for the measurement of complex matrices, such as human ETDA plasma, are given for the developed Simoa PCT assay even in the low pg·mL^−1^ range. Compared to the B·R·A·H·M·S PCT-sensitive Kryptor assay, the Simoa PCT assay achieved comparable performance with approximately a 50-fold higher sensitivity, which will make it possible to even detect PCT in healthy human individuals.

### 3.2. Correlation with Clinical Analyzer (B·R·A·H·M·S PCT-Sensitive Kryptor)

**Figure 2:**
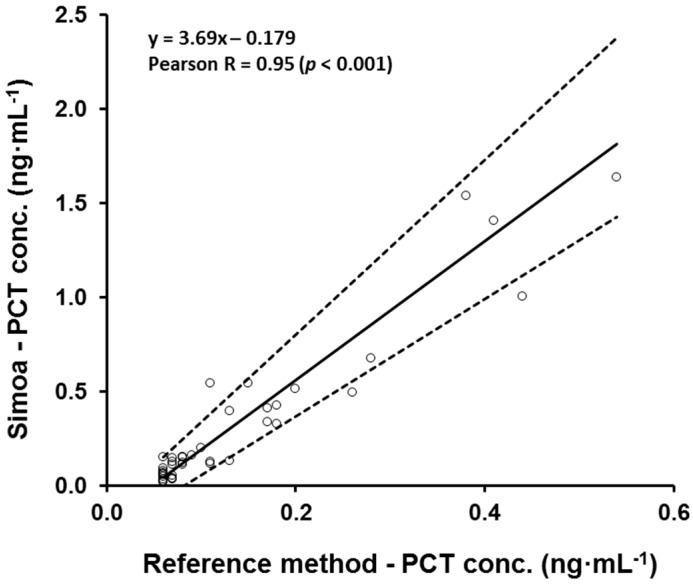
Passing–Bablok regression analysis of the PCT concentration of 42 samples obtained with the B·R·A·H·M·S PCT-sensitive Kryptor reference method and the Simoa PCT assay. Scatter diagram with regression line and 95% confidence bands for the regression line. Pearson correlation coefficient (R) of 0.95 (*p* < 0.001). Passing–Bablok regression line equation: y = 3.69x − 0.179 (intercept 95% confidence interval (CI): −0.255 to −0.125; slope 95% CI: 3.11 to 4.63).

To estimate the accuracy and systematic errors of the Simoa PCT assay, a comparison of methods was performed. Eighty one human samples, serum and CSF, were analyzed by the Simoa PCT assay and the B·R·A·H·M·S PCT-sensitive Kryptor reference method at the university medical center of Tuebingen. The B·R·A·H·M·S PCT-sensitive Kryptor assay is considered the gold standard PCT assay. To cover the working range of the Simoa PCT assay, samples with expected high, mid, and low PCT concentrations were analyzed by both methods (Serum and CSF samples from meningitis patients). The comparison of the two methods was performed via Passing–Bablok regression [18]. Thirty nine samples were excluded from the analysis because the PCT concentration of these samples was below the quantification limit (<0.06 ng·mL^−1^) of the Kryptor clinical analyzer, but could all be measured with the Simoa assay. The PCT level determined with the Simoa PCT assay was positively correlated with the PCT levels obtained with the clinical analyzer, resulting in a Pearson correlation coefficient (Pearson R) of 0.95 (two-sided test, *p* < 0.001). The equation of the Passing–Bablok regression line was y = 3.69x − 0.179 with a 95% confidence interval (CI) for slope from 3.11 to 4.63 and for intercept from −0.255 to −0.125. The 95% CI for the intercept did not include the value 0 and the 95% CI for the slope did not include the value 1. These results indicate a constant (intercept) and a proportional (slope) difference between the two methods for concentrations above 0.06 ng·mL^−1^ (quantification limit of the clinical analyzer). To adjust the results obtained with the Simoa PCT assay, a correction of the proportional difference was implemented. After back-calculation of the sample PCT concentrations obtained with the Simoa PCT assay, all values were corrected by dividing the results with the slope value (3.69). A correlation for PCT concentrations below 0.06 ng·mL^−1^ could not be performed, because of the lower sensitivity of the reference method compared to the Simoa PCT assay. To perform a correction of the constant difference between the two methods, the performance of a correlation for PCT concentrations below 0.06 ng·mL^−1^ would be mandatory because of the higher influence on the back-calculated PCT concentrations with lower PCT levels compared to concentrations above 0.06 ng·mL^−1^. For that reason, a correction of the constant difference was not performed. A Bablok regression scatter diagram with regression line and 95% confidence bands of the determined Simoa PCT concentrations and the reference values measured with the clinical analyzer is shown in Figure 2.

### 3.3. Baseline PCT Concentration in Healthy Volunteers

A first goal of this study was the determination of baseline PCT levels in healthy humans to establish physiological ranges which are not possible with the current gold standard methods due to limited sensitivity. PCT concentration was determined with the Simoa PCT assay in 90 samples from healthy donors, 41 serum and 49 EDTA plasma samples, respectively. CSF samples of healthy donors were not available, and therefore no baseline values could be established. Detailed information of the donors is summarized in Table S2. The samples of four donors were excluded from the analysis (two serum and two EDTA plasma samples). These four samples had PCT levels above 0.05 ng·mL^−1^, which is a commonly used and accepted cut-off for the discrimination of diseased and healthy patients. Such an increased PCT level can be due to a not yet diagnosed infectious disease, and therefore these donors were not considered as normal healthy control samples.

**Figure 3:**
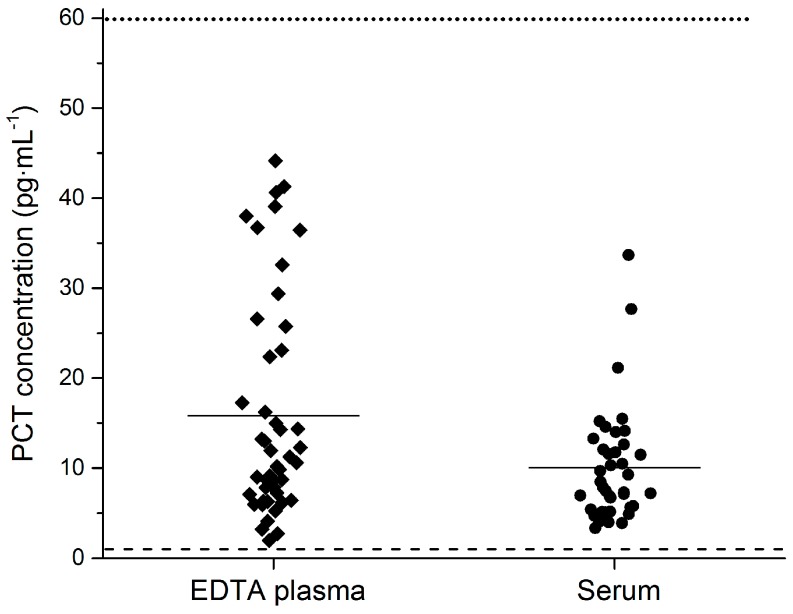
PCT concentration in healthy volunteers. Average PCT concentration in human K2 EDTA plasma and serum was 15.8 pg·mL^−1^ and 10.6 pg·mL^−1^, respectively. The PCT concentration of all 86 analyzed samples was above the LLoQ of the Simoa PCT assay. Data are presented as scatter plots. The middle line represents the mean value of a group, the dotted line the LLoQ of the B·R·A·H·M·S PCT-sensitive Kryptor assay, and the dashed line the LLoQ of the Simoa PCT assay.

The average PCT concentration in the 86 healthy donors was 13.2 pg·mL^−1^ (SD: 10.3). The average PCT concentration in the EDTA plasma and serum was 15.8 pg·mL^−1^ (SD: 12.2) and 10.6 pg·mL^−1^ (SD: 6.8), respectively. The PCT concentration in all 86 measured samples was above the limit of quantification of the Simoa PCT assay, which enabled a baseline PCT level determination of healthy individuals. For verification of the observed baseline levels, this should be done with a much larger donor cohort. A scatter plot of PCT levels in healthy donors with the LLoQ value lines of the Simoa PCT and the B·R·A·H·M·S PCT-sensitive Kryptor assay is presented in Figure 3.

### 3.4. Meningits Samples

Another goal of this study was to evaluate if the determination of PCT in the lower pg·mL^−1^ range could improve the diagnostic value of PCT in patients with bacterial or viral meningitis. PCT concentration was determined in the serum and cerebrospinal fluid (CSF) of 45 patients: 15 patients with diagnosed bacterial meningitis (BM), 15 patients with diagnosed viral meningitis (VB), and 15 patients of a control group. The diagnosis of meningitis was based on the clinical characteristics and laboratory findings of the subjects (Table S1).

To analyze statistical significance of the measured PCT concentrations between the groups, Mann–Whitney U tests (two-sided, significance level: 0.05) were performed. Results are reported as median and interquartile ranges (IQR).

**Figure 4:**
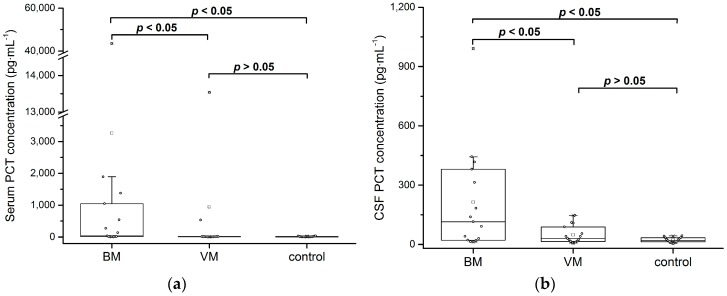
Comparison of PCT levels between patients with bacterial meningitis (BM), viral meningitis (VM), and control patients. PCT concentration was above the Simoa PCT assay LLoQ in all 90 measured samples. (**a**) PCT concentration in human serum. Statistically significant difference between the BM group and the VM and control groups (two-sided Mann–Whitney U test: *p* < 0.05). No statistically significant difference between the VM and control groups (two-sided Mann–Whitney U test: *p* > 0.05). (**b**) PCT concentration in human cerebrospinal fluid (CSF). Statistically significant difference between the BM group and the VM and control groups (two-sided Mann–Whitney U test: *p* < 0.05). No statistically significant difference between the VM and control groups (two-sided Mann–Whitney U test: *p* > 0.05).

The median CSF PCT concentration in the BM, VM, and control groups was 115 pg·mL^−1^ (IQR: 359), 29.9 pg·mL^−1^ (IQR: 73.9), and 18.8 pg·mL^−1^ (IQR: 20.7), respectively. The median CSF PCT level in the BM group was significantly higher in comparison to the VM (*p* = 0.042) and the control groups (*p* = 0.004). The median serum PCT concentration in the BM, VM, and control groups was 35.8 pg·mL^−1^ (IQR: 1032), 10.5 pg·mL^−1^ (IQR: 7.89), and 7.9 pg·mL^−1^ (IQR 12.5), respectively. Despite the unexpected low median concentration for the BM group, which is below the commonly used cut-off of 0.05 ng·mL^−1^, the median serum PCT level in this group was significantly higher in comparison with the VM (*p* = 0.004) and control groups (*p* = 0.001). In future work, this finding needs to be verified with a larger control group. PCT is known to be a marker for the detection of bacterial meningitis in CSF and serum [19,20,21,22]. This could be confirmed with the Simoa PCT assay. The median CSF and serum PCT level in the VM group was not significantly higher compared to the control group (both *p* > 0.05). Box plot diagrams with median lines, 25th and 75th percentile boxes, and 1.5·IQR error bars are presented in Figure 4.

PCT concentrations above 0.05 ng·mL^−1^ indicate an elevated PCT level and therefore a potentially infectious disease, such as bacterial meningitis. PCT levels below 0.05 ng·mL^−1^ were not measurable with commonly used methods and could not be determined; however, this is possible with the Simoa PCT assay. With this technology, the measured PCT concentrations in patients with bacterial and viral meningitis could be analyzed and differ significantly. The higher sensitivity of the Simoa PCT assay allows for the detection of PCT in the low pg·mL^−1^ range, and could be a method to detect PCT level elevations earlier and help to improve the monitoring of bacterial infectious diseases such as meningitis. The medical relevance of PCT levels below the clinical concentration range has to be evaluated, and the Simoa PCT assay is a possible sensitive tool for this application.

## 4. Conclusions

An ultrasensitive PCT immunoassay was developed on the Simoa platform. The assay was validated using a fit-for-purpose approach and under consideration of the recommendations for assay validation given in guidelines from health authorities (e.g., EMA, FDA) as far as applicable to ligand–binding assays. The developed assay fulfilled acceptance criteria for all addressed validation parameters, and validation demonstrated an improvement in sensitivity by a factor of 50 compared to the PCT gold standard assay, the B·R·A·H·M·S PCT-sensitive Kryptor assay. The increased sensitivity allowed for the determination of PCT in human serum and EDTA plasma in healthy volunteers, and therefore enabled us to establish a PCT baseline level in these matrices. Additionally, the Simoa PCT assay may be used to measure baseline PCT levels in other matrices, such as CSF. Both serum and CSF PCT showed significantly elevated PCT levels in patients with bacterial meningitis compared to patients with viral meningitis and the control group, as expected. PCT concentration levels for patients with viral meningitis and the control group could be measured, but were not significantly different. The determination of PCT in the low pg·mL^−1^ range could help to improve the monitoring of bacterial infectious diseases, as PCT level changes could be detected earlier. The medical relevance of PCT levels below the clinical concentration range has to be evaluated, and the ultrasensitive Simoa PCT assay is a possible sensitive tool for this application.

## Figures and Tables

**Table 1:** Validation results and acceptance criteria.

Validation Parameter	Acceptance Criteria	Results	Conclusion
Calibration model	80–120% recovery CV < 20%	four-parameteric logistics (1/Y^2^) 99–101% recovery CV < 20%	Complies
ULoQ	80–120% recovery CV < 20%	900 pg·mL^−1^ 102% recovery CV 5.0%	Complies
LLoQ	80–120% recovery CV < 20%	1.23 pg·mL^−1^ 101% recovery CV 15.3%	Complies
LoD (pg·mL^−1^)	zero calibrator +3× SD	0.44 pg·mL^−1^	
Intra-assay precision	mid-range: CV < 15% high-/low-range: CV < 20%	CV 13.5% CV 12.9–15.3%	Complies
Inter-assay precision	CV < 20%	CV 10.7–19.4%	Complies
Recovery	80–120% recovery	90–99% recovery	Complies
Dilutional linearity	80–120% recovery	82–117% recovery	Complies
Parallelism	80–120% recovery	91–115% recovery	Complies
Analyte stability	80–120% recovery	83–105% recovery	Complies

CV: coefficient of variation; ULoQ: upper limit of quantification; LLoQ: lower limit of quantification.

## Supplementary Materials

The following are available online at www.mdpi.com/2571-5135/6/4/18/s1, Table S1: Clinical characteristics and laboratory findings of the subjects for diagnosis of meningitis, Table S2: Sample list healthy volunteers purchased from Seralab.
