# Supplementary material for: Development and Validation of an Ultrasensitive Procalcitonin Sandwich Immunoassay
Source: High Throughput. 2017 Nov 16;6(4):18. doi: 10.3390/ht6040018 (PMC5748597; doi:10.3390/ht6040018)
Supplement: Supplementary file 1 [file high-throughput-06-00018-s001.pdf]

Supplementary Materials:

Development and Validation of an ultra-sensitive Procalcitonin sandwich immunoassay

Table S1. Clinical characteristics and laboratory findings of the subjects for diagnosis of meningitis

		Cell count				Albumin Quotient				Lactate			
**Diagnosis**	**N**	**Average**	**SD**	**Min.**	**Max.**	**Average**	**SD**	**Min.**	**Max.**	**Average**	**SD**	**Min.**	**Max.**
CSF - bakterial	15	350	810	5	3226	33.3	50.2	5.8	196	5.1	5	1.5	14.6
CSF - viral	15	93	121	2	373	8.7	6.4	3.4	28.4	1.9	0.4	1.5	2.7
CSF - control	15	1	1	0	4	4.9	1.8	3	9.9	1.7	0.4	1.3	2.3

Table S2. Sample list healthy volunteers purchased from Seralab

**Variable**	**EDTA plasma (*n* = 49)**	**Serum (*n* = 41)**
Age (years)	34 (SD 11)	37 (SD 11)
Age range (years)	19 - 59	18 - 67
Gender (male/female)	24 / 25	18 / 23
Race (black/caucasian/hispanic)	29 / 3 / 17	27 / 5 / 9
